# Fine-Scale Haplotype Mapping Reveals an Association of the *FTO* Gene with Osteoporosis and Fracture Risk in Postmenopausal Women

**DOI:** 10.3390/genes15091152

**Published:** 2024-09-01

**Authors:** Daniela Greere, Sara Haydar, Florin Grigorescu, Dana Manda, Gabriela Voicu, Corinne Lautier, Catalina Poiana

**Affiliations:** 1 Department of Endocrinology, C. I. Parhon Institute of Endocrinology, Carol Davila University of Medicine and Pharmacy, 011863 Bucharest, Romania; endoparhon@gmail.com; 2 Novo Nordisk Foundation Center for Basic Metabolic Research, Faculty of Health and Medical Sciences, University of Copenhagen, DK-2200 Copenhagen, Denmark; sara.haydar@sund.ku.dk; 3 Institut Convergences Migrations, 93322 Paris-Aubervilliers, France; 4 Molecular Cellular and Structural Endocrinology Laboratory, C. I. Parhon Institute of Endocrinology, 011863 Bucharest, Romania; dana.manda@gmail.com; 5 Nuclear Medicine Laboratory, C. I. Parhon Institute of Endocrinology, 011863 Bucharest, Romania; voicugabi@gmail.com; 6 Qualisud, Univ Montpellier, Avignon Université, CIRAD, Institut Agro, IRD, Université de La Réunion, 15 Ave Charles Flahault, 97400 Montpellier, France; corinne.lautier@umontpellier.fr

**Keywords:** *FTO*, osteoporosis, SNV, gene, haplotype

## Abstract

Introduction. The Fat Mass and Obesity-Associated (*FTO*) gene encodes a demethylase, which modulates RNA N6-methyladenosine (m6A) and plays a regulatory role in adipocyte differentiation and the pathogenesis of human obesity. Methods. To understand the potential role of *FTO* in osteoporosis (OP), we investigated five single nucleotide variations (SNVs) in intron 1 (rs8057044, rs8050136, rs9939609, rs62033406, and rs9930506) of the *FTO* gene, and a missense SNV i.e., rs3736228 (A1330V), located in exon 18 of the *LRP5* gene, in a cohort of postmenopausal women (*n* = 188) from Central Europe. Genotyping was performed with an allele discrimination assay, while haplotypes were reconstructed in the population by PHASE 2.1. Results. The rs9930506 was strongly associated with OP (*p* < 0.0035), which was supported by Bonferroni correction (*p* < 0.0175), and all SNVs located in the *FTO* gene were more strongly associated with severe OP with fragility fractures. Among seventeen haplotypes detected for the *FTO* gene, two haplotypes (H1 and H9) were frequent (frequency > 10%) and distributed in three main haplotypes pairs (H1/H1, H1/H9 and H9/H9, respectively). The pathogenic pair H1/H9 was associated with a leaner phenotype, increased fracture risk, and a lower bone mineral density (BMD), and carried the heterozygous GA of rs9930506, while the protective pair H9/H9 was associated with an increased obesity risk and carried AA alleles of rs9939609. Conclusions. Concordant associations with OP, an increased fracture risk, and a lower BMD at all skeletal sites indicate that the *FTO* gene is a promising candidate for OP, explaining the complex relationship with obesity and offering new perspectives for the study of the epigenetic regulation of bone metabolism.

## 1. Introduction

Osteoporosis (OP) is characterized by reduced bone mass and strength and altered bone architecture, predisposing individuals to fragility fractures [1]. With a prevalence of 20.5% in Central Europe (Romania), this disease is emerging as a pressing public health concern, particularly among the elderly population, due to the progressive deterioration of metabolic health, including the emergence of obesity, type 2 diabetes mellitus (T2D), and metabolic syndrome (MetS) [2,3]. The interplay between obesity and OP is complex, since obese women, through mechanical loading and estrogen production by the adipose tissue, exhibit a higher bone mineral density (BMD) with a potential protective role. The increased BMD does not necessarily translate to reduced fracture risk, a phenomenon known as the “obesity paradox” [4]. Among numerous factors that might contribute to an elevated risk of fragility fractures, genetic predisposition plays a determining role. A bivariate meta-analysis of a large-scale genome-wide association study (GWAS) indicated three loci (2p23.2, 16q12.2, and 18q21.32) with pleiotropic effects on both obesity and OP, corresponding to the TRNA Methyltransferase 61B (*TRMT61B*), *FTO*, and Melanocortin 4 Receptor (*MC4R*) genes, respectively [5]. Following the initial discovery of *FTO*’*s* implication in human obesity, this gene has garnered significant attention in other metabolic diseases such as T2D, non-alcohol fatty liver disease (NAFLD), hypertension, cardiovascular diseases, and OP [6,7]. 

The *FTO* gene encodes a Fe(II) and 2-oxoglutarate-dependent oxygenase that plays a crucial role in epigenetic regulation and functions as a demethylase of mRNA, specifically targeting m6A, which is the most prevalent RNA modification [8,9]. Studies involving cell cultures and animal models have demonstrated *FTO*’*s* involvement in adipogenesis, adipocyte apoptosis, and the osteogenic differentiation of bone marrow mesenchymal stem cells (BMSCs) into adipocytes or osteoblasts [9]. Apart from its expression in the adipose tissue, brain, muscle, and heart, *FTO* is also expressed in the bone marrow, rendering it a promising candidate for genetic predisposition to OP [10]. 

Although investigations into *FTO*’*s* role in OP in humans, and particularly postmenopausal women, have been limited, previous studies have identified intriguing associations. For instance, Guo et al. (2011) identified six single nucleotide variations (SNVs) in intron 8 linked to increased hip BMD in Chinese populations, although this finding was not replicated in a Caucasian sample [11]. Similarly, recent studies have reported several SNVs in intron 1 of *FTO* associated with hip fractures, albeit without an impact on BMD or bone loss rate [12,13].

The objective of this study was to shed light on the potential involvement of the *FTO* gene in postmenopausal OP by analyzing five common SNVs in intron 1, known from previous studies for their implications in human obesity and/or insulin resistance. Our analysis of unphased DNA revealed associations between SNVs and both OP and severe OP with fractures. Fine-scale haplotype mapping using phased DNA unveiled stronger association signals with OP and fragility fractures carried by specific haplotype combinations, aligning with the observed decrease in BMD at various skeletal sites.

## 2. Materials and Methods

### 2.1. Population

A total of 188 postmenopausal women were recruited at the C.I. Parhon National Institute of Endocrinology (Bucharest, Romania) from 28 May 2020 to 1 April 2022 [14]. The inclusion criteria were as follows: (1) women aged 50–75 years; (2) time from menopause ≥ 1 year; (3) Caucasian (*Romanian*) origin. Excluded were all forms of secondary OP after hormonal examination, as well as severe chronic diseases (except for T2D). The Institutional Ethical Committee approved the research protocol, and signed informed consent was obtained from each patient in accordance with the Helsinki Declaration [15].

Association was performed by comparing non-OP subjects (controls) and OP subjects (cases) classified according to the American Association of Clinical Endocrinologists (AACE) [16]. Criteria in postmenopausal women were based on any of the following: (1) T-score −2.5 or below in the lumbar spine, femoral neck, total proximal femur, or 1/3 radius; (2) low-trauma spine or hip fracture, regardless of BMD; (3) T-score between −1.0 and −2.5 and fragility fractures of proximal humerus, pelvis, or distal forearm; and (4) T-score between −1.0 and −2.5 and high FRAX fracture probability based on country-specific thresholds.

A comprehensive series of clinical, biochemical, and hormonal tests were performed (Table S1) as described previously [14]. All patients were examined by dual X-ray absorptiometry analysis (DEXA), and investigated for skeletal alterations and muscular performance (Table S2). A FRAX PLUS trabecular bone score (TBS) for the evaluation of the 10-year risk for low-energy fractures was computed on the country-specific website (https://www.fraxplus.org/ accessed on 16 July 2023). In addition, severe OP was diagnosed based on World Health Organization (WHO) criteria, namely the association of a T score equal to or less than −2.5 SD (e.g., <−3) with fragility fractures [17,18,19].

MetS diagnosis was based on the presence of at least three of the harmonized criteria of the National Cholesterol Education Program (NCEP) and the Adult Treatment Panel-III (ATP-III), which include (1) abdominal obesity based on waist circumference (WC) ≥ 88 cm, (2) high triglyceride (TG) levels ≥ 1.7 mmol/L, (3) low high-density lipoprotein cholesterol (HDL-C) < 1.03 mmol/L, (4) high blood pressure (HBP) with systolic blood pressure (SBP) ≥ 130 mmHg and/or diastolic blood pressure (DBP) ≥ 85 mmHg, and (5) high fasting glucose levels ≥ 5.36 mmol/L, or current treatment with antihyperlipidemic, antihypertensive, or hypoglycemic agents, respectively [20]. Insulin resistance was assessed using homeostasis model assessment (HOMA-IR) or as a nominative variable defined as having HOMA-IR values above the cutoff of 1.92, which was calculated from fasting insulin levels of lean patients without OP + 2 SEM, as previously described [21]. To resume the evaluation of muscular strength and physical performance, we created a statistical instrument (SUM^stat^) to consider 0, 1, 2, 3, 4, or all 5 muscular tests outside the normal values, using a binary (0/1) parameter (Table S2).

### 2.2. Genotyping

Genomic DNA was extracted from whole blood using the Wizard Genomic DNA Purification Kit (Promega, Madison, WI, USA) as described previously [21]. Five SNVs in intron 1 of the *FTO* gene (rs8057044, rs8050136, rs9939609, rs62033406, and rs9930506) were selected based on our previous studies, particularly regarding the linkage disequilibrium (LD) block around rs9939609, to which we added some other validated SNVs from the Affymetrix database [22,23,24,25]. We also genotyped one missense SNV, rs3736228 (A1330V), in the *LRP5* gene identified from GWAS data. Their positions were referenced for GRCh37/hg19, and the nomenclature was validated using the VariantValidation site (https://variantvalidator.org/ accessed on 1 June 2024). Genotyping was performed with an allele discrimination assay (KASPar technique from LCG Genomics, Teddington, UK). For phased DNA, haplotypes were reconstructed in the population using the PHASE 2.1 program [26] and visualized for LD in HAPLOVIEW 3.1 [27], while predictions of transcriptional activity were examined in HaploReg v4.1 (http://archive.broadinstitute.org/mammals/haploreg, accessed on 1 June 2024).

### 2.3. Statistics and Computation

Statistical analysis was performed using the StatView 5.0 program (Abacus Concepts, Berkeley, CA, USA), and the study was powered at 0.85 using PBAT, as described previously [22]. Numerical variables expressed as mean ± standard errors of the mean (SEM) were tested by non-parametric Kruskal–Wallis and Mann–Whitney U tests. For the ANOVA, interaction factor α was set at 5%. Nominal variables were analyzed using the χ^2^ test, and logistic regression was performed with the descent method to obtain *p*-values, odds ratios (ORs), and 95% confidence intervals (CIs). Significance was considered at *p* < 0.05. Bonferroni corrections for the genetic association of SNVs were performed in the R v3.2.1 program. The LD among SNVs was calculated in the NIH database (https://ldlink.nih.gov accessed on 1 June 2024) and in HAPLOVIEW 3.1. Genotype–phenotype correlation was performed in the OP population, and the results for haplotype pairs were correlated with those from independent SNVs on unphased DNA. For BMD, all anatomical sites were tested, and when indicated, values were adjusted for body mass index (BMI).

## 3. Results

### 3.1. SNP Association

The phenotypic features of OP and controls have been described in detail previously [14]. Briefly, women with OP were on average 66.4 ± 0.7 years old, with a BMI of 26.2 ± 0.4 kg/m^2^, mean ± SEM), with 20.8% being classified as obese and 32% showing insulin resistance. MetS was diagnosed in 48.3% of cases. Fragility fractures were detected in 48.3% of OP cases. Control women were more obese (BMI of 30.6 ± 0.6 kg/m^2^), with a higher proportion of MetS (51.5%) but with a comparable level of insulin resistance (35.3%) based on the HOMA-IR index. Five SNVs in the *FTO* gene (rs8057044, rs8050136, rs9939609, rs62033406, and rs9930506) and one SNV in the *LRP5* gene (rs3736228) had a minor allele frequency (MAF) comparable to Europeans and were in the Hardy–Weinberg equilibrium. Using an over-dominant model in logistic regression, rs9930506 (GA) was associated with OP with a high OR, while the association of rs8057044 (GA) and rs9939609 (TA) appeared as a trend (Table 1). 

The association of rs9930506 was supported by the Bonferroni correction (*p* < 0.0175). No association was detected for rs3736228 in the LRP5 gene, which was not further studied. All five SNVs were significantly associated with severe OP with fractures, and among these, rs9939609 had a protective effect. Genuine associations with severe OP were sustained by Bonferroni correction for rs8057044 (*p* < 0.013) and again for rs9930506 (*p* < 0.001). Conditional analysis showed that rs9930506 and rs9939609 had independent associations (*p* < 0.0001 and 0.01, respectively) in the corresponding LD block. Of note, the SNVs rs8057044 and rs9930506 were in reduced LD with r^2^ = 0.70 (https://ldlink.nih.gov). Therefore, for further correlation with the biological parameters, we considered rs8057044, rs9939609, and rs9930506 as lead SNVs.

### 3.2. Haplotype Mapping

**Figure 1:**
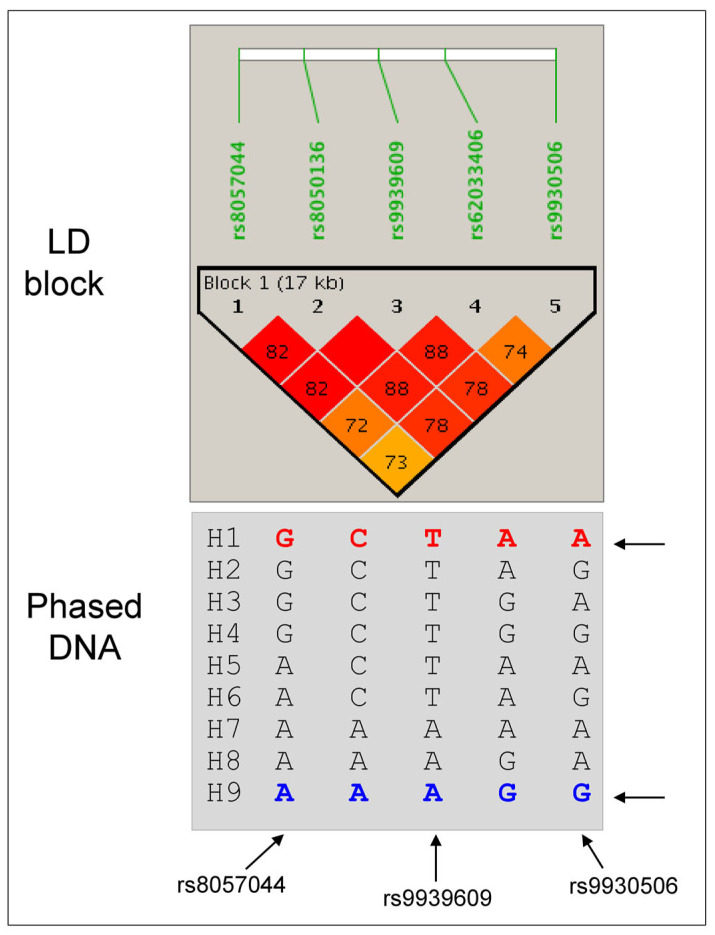
LD map of SNVs in intron 1 of the *FTO* gene, and haplotypes reconstructed with the PHASE program.

To further understand the biological effects of the SNVs, we performed haplotype mapping using the PHASE program. A total of nine haplotypes were reconstructed in the population, which were then assigned to dizygotic individuals as haplotype pairs (Figure 1).

Two haplotypes (H1 and H9) were more prevalent (>40%), while others (H2 to H8) were rare or very rare. Haplotype H4 was absent in controls, while haplotypes H6 and H7 were absent in severe OP, which displayed only haplotype H8. There were seventeen haplotype pairs in the population, among which three pairs were frequent: H1/H1 (24.5%), H1/H9 (35.6%), and H9/H9 (20.2%). All other pairs had a frequency less than 5%. When tested independently, only one rare haplotype (H6) showed a trend association with OP (*p* < 0.0557), which was protective.

A completely different picture emerged from the analysis of haplotype pairs. While the H1/H1 pair remained non-significant, H9/H9 was associated with severe OP with a protective effect. By contrast, the heterozygous H1/H9 pair was associated with both OP and severe OP with a high OR (Table 1). The H1/H9 association was stronger in insulin-resistant individuals with an OR of 3.92, 95% CI [1.48–10.367], and *p* < 0.0029.

To understand the metabolic consequences, variations in OR were examined as a function of the presence or absence of MetS or its components. When the population was stratified as with or without MetS, a significant association was detected for H1/H9 in the absence of MetS (*p* < 0.03, OR 1.9, 95% CI [1.043–3.626]) and the absence of low HDL levels (*p* < 0.0057, OR 2.3, 95% CI [1.249–4.242]) in individuals with HBP with *p* < 0.0093, OR 2.5, and 95% CI [1.228–5.148], and particularly in women with high TG levels (*p* < 0.0079, OR 4.58, 95% CI [1.35–15.98]). The same picture was obtained for the H1/H9 association in the sub-population of OP with fractures, with the OR for the H1/H9 pair being increased up to 9.1 (*p* < 0.0067) in individuals with high TG levels. These data concordantly indicated that the H1/H9 haplotype pair was pathogenic for OP and fractures, contingent upon the presence of insulin resistance with high TG levels and HBP rather than obesity, low HDL levels, hyperglycemia, or the presence of MetS.

### 3.3. Genotype–Phenotype Correlation

To search for metabolic consequences and bone alterations, we examined the genotype–phenotype correlation in the OP population by investigating the impact of the three most frequent haplotype pairs, and correlated the results with those from the independent unphased DNA lead SNVs rs8057044, rs9939609, and rs9930506 (Tables S3–S5).

Carriers of H1/H9 haplotypes exhibited a leaner phenotype, attributed to the heterozygous GA, TA, or GA genotypes of rs8057044, rs9939609, and rs9930506, respectively (Table 2). Despite their leaner phenotype (12.2% obesity), this SNV combination was found to be pathogenic for bone, being associated with 53% fractures and the highest prevalence of muscular alterations (51%). The FRAX scores were also the highest.

By contrast, homozygous H9/H9 carriers with AA, AA, and GG alleles of the three lead SNVs, respectively, were more obese (30.2% obesity), with the highest WC (97.2 cm), central obesity (86%), and SBP. In unphased DNA, these SNVs displayed a higher BMI and higher prevalence of obesity, larger WC, and central obesity, features that may stem from the effect of the homozygous AA allele of rs9939609, extensively studied in human obesity [28]. 

Finally, H1/H1 homozygous carriers of GG, TT and AA alleles of the lead SNVs exhibited an intermediate phenotype combining GG alleles of rs8057044 (influential in obesity) as well as the TT and AA alleles of rs9939609 and rs9930506, respectively (associated with a leaner phenotype). No effects of haplotype pairs were found on the HOMA-IR index, insulin resistance as a nominative variable, fasting glycemia, or MetS, although in H1/H1 carriers there was a trend towards a higher prevalence (39.3%) of MetS and low levels of HDL (46.4%, *p* < 0.0003). These latter effects may be driven by the dominant effect of the rs9930506 AA genotype.

**Figure 2:**
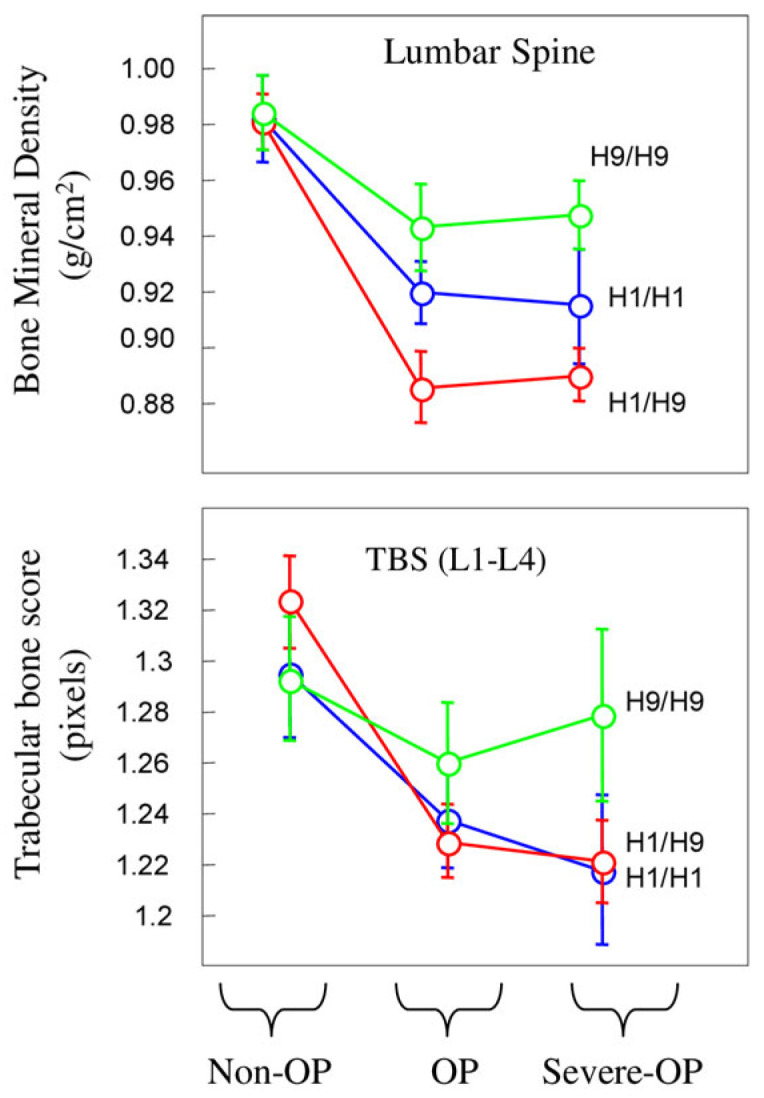
Variations in BMD (lumbar spine) and TBS as function of haplotype pairs in non-OP, OP, and severe OP populations.

Next, our focus shifted to bone metabolism, and in particular BMD, TBS, and bone turnover markers (Table 2). Carriers of the pathogenic pair H1/H9 exhibited lower BMD values at all anatomical sites, while H9/H9 revealed higher values, as shown in Figure 2 for the lumbar spine.

Among anatomical sites, the radius demonstrated the most significant difference (*p* < 0.0001). Although TBS values were lower compared to the controls, they did not reach statistical significance (*p* < 0.0501). However, significance was obtained for GA of rs8057044 (*p* = 0.0192), AA of rs9939609 (*p* = 0.0135), and GG of rs9930506 (*p* = 0.0453) when independent SNVs were considered in unphased DNA.

For bone turnover markers, only Beta-crosslaps showed higher values in the H9/H9 pair. Additionally, there was a trend towards a higher prevalence of muscular alterations (51.0%) in H1/H9 carriers compared to H1/H1 and H9/H9 (32.1% and 46.3%, respectively), with the significance obtained only for reduced grip strength (right hand). Carriers of the H1/H9 pair exhibited higher values for FRAX PLUS major and hip fracture risks (9.8 and 3.2, respectively), with significance between H1/H9 and H9/H9 carriers (Table 2 and Table S6).

## 4. Discussion

In this paper, we present evidence for the association of SNVs in intron 1 of the *FTO* gene with OP and severe OP with fragility fractures, designating this gene as a promising candidate for OP. An association was observed using unphased independent SNVs and, in addition, haplotype mapping indicated stronger association signals and correlated well with biological parameters of bone metabolism, particularly BMD. These data reinforce previous studies on the *FTO* gene and, through haplotype mapping, provide new data useful in the description of potential biomarkers for the genetic predisposition for OP in postmenopausal women.

We studied five SNVs in intron 1 of the *FTO* gene selected from our previous investigations into human obesity and MetS in French, Romanian, and North African populations and extensively studied in human obesity [21,22,23,24,25,28]. We focused particularly on the LD block around rs9939609 [25] and added some new SNVs from our Affymetrix database created during the MEDIGENE program (FP7 279171-1), as described previously [22]. The rs1421085 was not genotyped in this study, as explained by a low LD with the other SNVs. Among the SNVs studied, rs8057044 is reported here for the first time as being associated with OP and fracture risk. This SNV displayed a low LD with the other four SNVs in intron 1, suggesting that it might contain a distinct signal. Except for rs8057044, the remaining four SNVs were already reported in relation to hip fractures but without a correlation with BMD [11,12,13]. The rs9939609 SNV was extensively studied as a marker for human obesity. One article reported rs9939609 as being associated with spine BMD (*p* = 0.037) in a Chinese population, although a protective or pathogenic role was not indicated, as the paper was focused on SNVs in intron 8 [11]. 

Two other studies in the Australian Dubbo Osteoporosis Epidemiology Study (DOES) collection investigated SNVs in intron 1 and found association with hip fractures, but again without an effect on BMD [12,13]. In these Australian studies, rs9930506 had the best association with hip fractures (HR of 2.19, *p* < 0.01), a result that is concordant with our best association for the same SNV in the Romanian population. The well-known obesity associated rs9939609 was non-significant (*p* < 0.21) in the Australian samples in association with hip fractures [12] while in our sample, the same SNV showed a more protective effect regarding OP, being linked with obesity. Finally, another SNV (rs17817712), located between rs9939609 and rs62033406 was identified by a meta-analysis of large-scale GWAS data as being associated with both obesity and OP. The data were confirmed by the UK Biobank, indicating the pleiotropic effect of *FTO* on both obesity and OP [5]. From all these data on independent SNVs in unphased DNA, including this study, we conclude that intron 1 contains a strong associated signal for OP and fracture risk, although we cannot exclude the possibility that intron 1 contains distinct signals, at least between rs8057044 and rs9930506 (pathogenic) and rs9939609 (protective), as a function of the concomitant association with obesity or lack thereof.

Our study further analyzed intron 1 in phased DNA through fine-scale haplotype mapping, and obtained better associations for OP and severe OP with fractures. Data were concordant with the decrease in BMD at all anatomical sites, including the lumbar spine, femoral neck, hip, and radius. These results were expected due to the detailed resolution of this genomic region [23,24,25]. Indeed, nine haplotypes obtained from five SNVs indicated a good resolution compared to the potential 2^5^ theoretical combinations of SNVs. While independent haplotypes were still not significant, haplotype pairs reached the highest OR for association. We focused on three major haplotype pairs (H1/H1, H9/H9, and H1/H9), among which H1/H9 was pathogenic in lean subjects. The effects on the biological parameters were concordant with those drawn from independent SNVs in unphased DNA. The H9/H9 pair was by contrast associated with a high BMI and obesity, containing the pathogenic AA alleles of rs9939609, while H1/H9, with GA, TA, and GA alleles of the lead SNVs, were associated with both leaner and obese phenotypes. These results suggest that there could be variability in OP phenotypes as a function of combinations of SNVs with synergistic or antagonistic effects. This might explain why the association of OP with rs9939609 was not always found in human studies, such as for instance in GWAS for OP.

All these data were corroborated with new results from the literature and indicate that the *FTO* gene, operating as a demethylase, is a promising candidate in the pathogenesis of OP. The mechanism is not completely understood. The *FTO* gene is expressed in numerous tissues, including the brain, adipose tissue, and bone marrow. It is very likely that demethylase activity involves a shift from differentiation to osteoblasts towards bone marrow adipocytes. It was recently shown that methylated m6A RNA levels are upregulated in the bone marrow in patients with OP. Moreover, there is experimental evidence that *FTO* overexpression in normal BMSC cells could compromise osteogenic potential, by decreasing methylated m6A and the level of runt-related transcriptional factor 2 (Runx2) mRNA [29]. Although the SNVs studied are involved in the demethylation process, the proteins bound are different: Nanog and Pou5f1 (rs9939609), P300 (rs8050136), and HMG-IY (rs62033406). rs8057044 involves Pax-5 and Rad21, while rs9930506 involves IRX proteins. We cannot exclude the possibility that the best associated SNV, rs9930506, operates in a similar mode to another upstream SNV (rs1421085), by binding to the AT-rich interaction domain 5B (ARID5B) repressor protein and the enhancer region of Iroquois Homeobox (IRX) 3 and 5, known to suppress the expression of *IRX3* and *5* [30]. As indicated above, rs1421085 was not investigated in this study, but we previously showed its role in simple and morbid obesity and its association with insulin resistance and MetS in Romanian subjects [23,24].

We were unable to find a direct relationship between SNVs and insulin resistance as measured by the HOMA-IR index, or with altered glycemic levels. This result was unexpected, since the OR of association was found to be higher when insulin resistance stratified the population. This might be explained by the multiple determinants of HOMA-IR values, including immunological alterations such as those involved in MetS [3]. Indeed, in the same population, we previously showed that the HOMA-IR was proportional to the cumulative criteria for MetS and not simply to BMI [14]. We cannot exclude the possibility that variations in insulin resistance parameters (e.g., HOMA-IR index) are too high for differences among genotypes to be detected, as indicated in Table S7 with the SD of values. We did not find an association of the SNV rs3736228 of the LRP5 gene, either, although there would be many other influential SNVs in this gene in OP, as indicated by the GWAS data. Further studies are necessary in order to understand the complex interplay among gene candidates in OP. 

The relationship between obesity and OP remains complex, involving not only systemic insulin resistance, but also other factors such as lifestyle (physical activity, smoking, alcohol consumption) and metabolic complications in the elderly population with OP. In this study, we presented the genuine association of SNVs and haplotypes or haplotype pairs, and the potential role of covariates was studied by a stratification of the population with and without MetS, insulin resistance, or the presence of low TG levels, which indicated a high variation in the OR. Stratification by age did not affect the genetic association of SNVS or haplotypes. In the same population, we previously reported that the relationship between BMI (expressed as percentiles) and BMD was not linear, and that the decrease in BMD in OP occurred beyond an inflection point of 27.2 kg/m^2^ of BMI [14]. A similar inflection point was found in other populations and correlated to the proportion of fat versus lean mass [31]. These recent observations suggest that some variability in *FTO* studies might be explained by the clinical classification of individuals as lean, overweight, or obese, as well as by different definition of MetS in different ethnic groups. Further studies in homogenous groups of patients with varying degrees of obesity would be necessary to understand the effect of *FTO* genetic variations on systemic insulin resistance and the specific effects on bone.

## 5. Conclusions

In conclusion, the strength of this paper is the identification of SNVs in intron 1 of the *FTO* gene that are robustly associated with primary OP and severe OP with fragility fractures and in concordance with the decrease in BMD at different anatomical sites. The study, performed among well-characterized postmenopausal women, albeit in a small size sample, indicates *FTO* as a promising gene candidate for OP, in which haplotype mapping in phased DNA offers new supplementary insights into the multifaceted association signals of the *FTO* gene with OP. 

## Figures and Tables

**Table 1:** SNV and haplotype of *FTO* gene association with OP and severe OP with fractures in Romanian population.

SNV or Haplotype ID ^a^	Allele or Sequence	Frequency (Entire Population)	Non-OP ^b^ (Ctr) (*n* = 68)	OP (Cases) (*n* = 120)	Severe OP (Cases) (*n* = 54)	*p* Value Ctr/OP	OR [95%CI]	*p* Value Ctr/Sev-OP ^c^ (Genotypes)	OR [95%CI]
SNVs (unphased DNA)									
rs8057044	G/A	0.49	0.39	0.42	0.59	0.0548	1.52 [0.99–2.33]	**0.0026** (GA)	2.2 [1.32–3.69]
rs8050136	C/A	0.44	0.42	0.37	0.57	0.4514	1.18 [0.77–1.49]	**0.0225** (CA)	1.81 [0.09–3.02]
rs9939609	T/A	0.44	0.42	0.37	0.57	0.0514	0.85 [0.56–1.29]	**0.0225** (TA)	0.55 [0.33–0.92]
rs62033406	A/G	0.46	0.42	0.39	0.57	0.3636	1.22 [0.79–1.86]	**0.0225** (GA)	1.81 [1.09–3.02]
rs9930506	A/G	0.48	0.35	0.44	0.60	**0.0035**	1.89 [1.23–2.92]	**0.0002** (GA)	2.66 [1.58–4.49]
Haplotypes (phased DNA)									
H1	GCTAA	0.46	0.43	0.49	0.46	0.3964	1.21 [0.79–1.83]	0.6493	1.12 [0.68–1.87]
H2	GCTAG	0.03	0.04	0.01	0.04	0.3656	0.56 [0.16–1.96]	0.9911	1.00 [0.26–3.84]
H3	GCTGA	0.02	0.02	0.01	0.02	0.7132	0.75 [0.15–3.41]	0.8457	0.83 [0.14–5.09]
H4	GCTGG	0.01	0.00	0.01	0.00	0.1795	NA	NA	NA
H5	ACTAA	0.03	0.02	0.02	0.05	0.5244	1.52 [0.39–5.86]	0.3016	2.15 [0.50–9.21]
H6	ACTAG	0.02	0.04	0.01	0.00	0.0557	0.22 [0.04–1.15]	NA	NA
H7	AAAAA	0.01	0.01	0.08	0.00	0.6891	0.56 [0.03–9.11]	NA	NA
H8	AAAGA	0.00	0.00	0.00	0.01	0.3429	NA	NA	NA
H9	AAAGG	0.43	0.44	0.42	0.046	0.7609	0.09 [0.61–1.43]	0.8113	0.94 [0.56–1.56]
Major haplotype pairs									
H1/H1	GCTAA GCTAA	0.24	0.26	0.23	0.18	0.2143	0.73 [0.45–1.19]	0.1441	0.63 [0.34–1.170]
H9/H9	AAAGG AAAGG	0.20	0.23	0.23	0.13	0.2316	0.73 [0.44–1.22]	**0.0385**	0.48 [0.24–0.96]
H1/H9	GCTAA AAAGG	0.36	0.26	0.35	0.48	**0.0047**	1.92 [1.21–3.06]	**0.0005**	2.58 [1.51–4.41]

^a^, SNVs have the following position in GRCh37/hg19: 53812614 (rs8057044), 53816275 (rs8050136), 53820527 (rs9939609), 53824226 (rs62033406), and 53830465 (rs9930506); ^b^, CTR stands for controls; ^c^, sev-OP stands for severe OP with fractures; genotypes tested in logistic regression (over-dominant model); significant values are indicated in bold. Alleles of three leader SNVs are underlined in haplotype sequences.

**Table 2:** Genotype–phenotype correlation of haplotype pairs of *FTO* gene in OP. Data are presented as mean ± SEM. Numerical variables were compared using the Kruskal–Wallis test (between three pairs) and Mann–Whitney U test (between two haplotype pairs), while nominal variables were tested by χ^2^. Significant values are in bold and trend values (0.05 < *p*-value ≥ 0.10) are in italics.

Parameter	Homozygous H1/H1	Homozygous H9/H9	Heterozygous H1/H9	*p*-Value ^a^ Kruskal–Wallis	*p*-Value Mann–Whitney	*p* ANOVA ^h^ (α)
Metabolic parameters						
Age (years)	65.5 ± 1.0	67.0 ± 1.2	65.9 ± 0.9	NS	NS	NS
BMI (kg/m^2^)	26.5 ± 0.6	28.1 ± 0.7	24.7 ± 0.5	**0.0002**	**0.0188** * 0**.0001** *	**0.0002** (0.983)
SBP ^b^ (mmHg)	119.8 ± 2.8	130.2 ± 3.0	120.7 ± 1.4	**0.0412**	**0.0017** **	**0.0057** (0.84)
DBP (mmHg)	76.4 ± 1.5	79.2 ± 1.9	74.1 ± 0.9	NS	**0.0069** **	**0.0277** (0.66)
HDL-C ^c^ (mmol/L)	1.4 ± 0.0	1.5 ± 0.0	1.5 ± 0.0	*0.0622*	**0.0184** * 0.0172 *	**0.0313** (0.64)
Central obesity (%)	71.42	86.04	55.10	**0.0011**	NA	NA
Low HDL-C (%)	46.42	9.30	28.57	**0.0003**	NA	NA
MetS^ATPIII^	39.28	18.60	28.56	*0.0789*	NA	NA
CRP (mmol/L) ^d^	38.1 ± 9.52	38.1 ± 0.0	28.6 ± 0.0	**0.0123**	**0.0298** *** 0.0747 *	*0.0586* (0.54)
Bone and muscular parameters						
Severe OP with fractures (%)	35.71	32.55	53.06	**0.0285**	NA	NA
BMD LS (g/cm^2^) ^e^	0.92 ± 0.01	0.94 ± 0.01	0.89 ± 0.01	**0.0003**	**0.0001** * 0.0208 *	**0.0002**
BMD HIP (g/cm^2^)	0.97 ± 0.01	0.99 ± 0.01	0.94 ± 0.01	**0.0002**	**0.0001** * 0.0188 ***	**0.0002** (0.98)
BMD FN (g/cm^2^)	0.80 ± 0.01	0.82 ± 0.01	0.79 ± 0.01	**0.0002**	**0.0001** ** 0.0188 ***	**0.0002** (0.98)
BMD RA (g/cm^2^)	0.64 ± 0.00	0.65 ± 0.00	0.63 ± 0.00	**<0.0001**	**0.0001** ** 0.0206 * 0.0423 ***	**<0.0001** (0.99)
Beta-crosslaps (ng/mL)	0.37 ± 0.03	0.70 ± 0.03	0.35 ± 0.02	NS	**0.0338** ** 0.0306 *	*0.0739* (0.51)
TBS L1–L4 (pixels) ^f^	1.23 ± 0.02	1.26 ± 0.02	1.22 ± 0.01	NS	*0.0501* **	NS
SUM^stat^ (%) ^g^	32.14	46.31	51.02	*0.0738*	NA	NA
Grip strength right (kg)	20.96 ± 0.56	22.57 ± 0.83	20.56 ± 0.45	NS	**0.0223** **	*0.0584*
FRAX PLUS hip (%)	2.69 ± 0.36	2.23 ± 0.19	3.28 ± 0.31	NS	**0.0326** **	NS

^a^. NS stands for non-significant and NA for non-applicable; ^b^. SBP and DBP stand for systolic and diastolic blood pressure. ^c^ HDL-C stands for High-density lipoprotein-cholesterol ^d^. CRP stands for C-reactive protein. ^e^. BMD for LS (lumbar spine), FN (femoral neck), and RA (33% radius). All BMD values were adjusted for BMI. ^f^. TBS, trabecular bone score. ^g^. SUM^stat^, summary statistics of muscular tests. ^h^. α stands for interaction factor. * indicates statistical significance between H1/H1 and H9/H9; ** indicates significance between H1/H9 and H9/H9; *** indicates significance between H1/H1 and H1/H9.

## Data Availability

Full data may be obtained from the authors upon request.

## Supplementary Materials

The following supporting information can be downloaded at: https://www.mdpi.com/article/10.3390/genes15091152/s1: Table S1: Clinical and biochemical assessments of OP; Table S2: Skeletal assessment, muscular strength, and physical performance; Table S3: Genotype–phenotype correlation of SNV rs8057044 (G/A) in OP; Table S4: Genotype–phenotype correlation of SNV rs9939609 (T/A) in osteoporotic patients; Table S5: Genotype–phenotype correlation of SNV rs9930506 (G/A) in OP; Table S6: Genotype–phenotype correlation of haplotype pairs of the *FTO* gene in OP. Table S7. Features of non-osteoporotic (non-OP) and osteoporotic (OP) patients.

## Abbreviations and Acronyms

AACE	American Association of Clinical Endocrinologists
ALP	Alkaline phosphatase
ATP-III	Adult Treatment Panel-III
BMD	Bone mineral density
BMI	Body mass index
BMSC	Bone marrow mesenchymal stem cells
CRP	C-reactive protein
CST	Chair stand test
DBP	Diastolic blood pressure
DEXA	Dual X-ray absorptiometry
ECLIA	Electrochemiluminescent immunoassay
HBP	High blood pressure
HDL-C	High-density lipoprotein cholesterol
HOMA	Homeostasis model assessment
LDL-C	Low-density lipoprotein cholesterol
GWAS	Genome-wide association study
LD	Linkage disequilibrium
MAF	Minor allele frequency
MetS	Metabolic syndrome
m6A	N6-methyladenosine
NCEP	National Cholesterol Education Program
NAFLD	Non-alcohol fatty liver disease
OP	Osteoporosis
P1NP	Procollagen type I N-terminal propeptide
PTH	Parathyroid hormone
SBP	Systolic blood pressure
SNV	Single nucleotide variation
T2D	Type 2 diabetes mellitus
TBS	Trabecular bone score
TG	Triglycerides
TUG	Timed up and go test
WC	Waist circumference
WHO	World Health Organization
