# Supplementary material for: Fine-Scale Haplotype Mapping Reveals an Association of the FTO Gene with Osteoporosis and Fracture Risk in Postmenopausal Women
Source: Genes (Basel). 2024 Sep 1;15(9):1152. doi: 10.3390/genes15091152 (PMC11431166; doi:10.3390/genes15091152)
Supplement: Supplementary file 1 [file genes-15-01152-s001.zip › genes-3132851-supplementary.pdf]

genes MDPI

# Supplementary Materials

## Table S1. Clinical and biochemical assessments of OP

| | **References** |
|---|---|
| Body weight, height, and *waist circumference* (WC) were recorded, and BMI was calculated as weight in kg divided by the square of height in meters. The cutoff of 30 $kg/m^2$ for BMI was used to define obesity. A comprehensive set of blood tests (after an overnight fast) was performed using a clinical chemistry autoanalyzer (Cobas c 501, Roche Diagnostics), including blood count, fasting glucose, urea, creatinine, uric acid, albumin, total protein, total cholesterol, high-density lipoprotein cholesterol (HDL-C), low-density lipoprotein cholesterol (LDL-C), triglycerides (TG), total and ionized calcium, magnesium, phosphate, alkaline phosphatase (ALP), erythrocyte sedimentation rate (ESR), C-reactive protein (CRP), and fibrinogen. Insulin, (25-OH) vitamin D, parathyroid hormone (PTH), osteocalcin, procollagen type I N-terminal propeptide (P1NP), and beta-Crosslaps were measured using electrochemiluminescent immunoassay (ECLIA) with a Cobas e601 module (Roche Diagnostics) [1] | 1. D. Greere, F. Grigorescu, D. Manda, G. Voicu, C. Lautier, I. Nitu and C. Poiana. Relative Contribution of Metabolic Syndrome Components in Relation to Obesity and Insulin Resistance in Postmenopausal Osteoporosis. J. Clin. Med. 13 (2024) 2529 – 2547. DOI: 10.3390/jcm13092529 |

## Table S2. Skeletal assessment, muscular strength and physical performance

| | **References** |
|---|---|
| **Skeletal assessment**. The population included women with normal bone mineral density (BMD), newly diagnosed or already treated osteopenia and primary OP. For postmenopausal women, normal BMD was defined as a T score between +1 and -1 SD, while osteopenia was defined as a T score between -1 and -2.5 SD. OP was defined according to the *American Association of Clinical Endocrinologists* (AACE) and *National Osteoporosis Foundation guidelines* [2]. We used specific AACE criteria in postmenopausal women based on any of the following: 1) T-score −2.5 or below in the lumbar spine, femoral neck, total proximal femur, or 1/3 radius; 2) Low-trauma spine or hip fracture, regardless of BMD; 3) T-score between −1.0 and −2.5 and fragility fractures of proximal humerus, pelvis, or distal forearm; 4) T-score between −1.0 and −2.5 and high FRAX® fracture probability based on country-specific thresholds. FRAX PLUS (TBS) score for the evaluation of a 10-year risk for low energy fractures was computed on the country-specific website (https://www.fraxplus.org/). In addition, severe OP was diagnosed based on World Health Organization (WHO) criteria, namely the association of T score equal to or less than -2.5 SD (e.g., < -3) and fragility fractures [3]. Patients with osteopenia and OP underwent lateral spine radiography, and additional prevalence of fragility fractures was established from the medical history. Bone micro-architecture was evaluated by *Trabecular Bone Score* (TBS), calculated for the lumbar spine at L1–L4.<br><br>**Muscular strength and physical performance**<br><br>The following tests were used:<br><br>**Handgrip strength** (HGS) was measured bilaterally using a calibrated digital handheld dynamometer (KERN MAP 80K1S) with the patient in a sitting position, the shoulder adducted and neutrally rotated, the elbow against the body, flexed at 90 degrees, and the forearm and wrist in a neutral position. Mean data of three trials are expressed in kg. Normal values are ≥ 16 kg [4].<br><br>**Chair Stand Test** (CST) was performed with a standard height chair with a straight back and no armrests and placed next to a wall. Each subject was instructed, at the count of "go," to stand up fully and sit down five times as fast as possible while keeping their arms folded across the chest and without resting on the chair in between repetitions. The patient was not talked during the test to avoid interfering with the patient's speed. The 5xSTS was timed from initial to final seated position and was expressed in seconds. Normal values are ≤ 15 sec [4]. | 2. Kanis, J.A.; Cooper, C.; Rizzoli, R.; Reginster, J.-Y. European Guidance for the Diagnosis and Management of Osteoporosis in Postmenopausal Women. Osteoporos. Int. 2019, 30, 3–44. https://doi.org/10.1007/s00198-018-4704-5<br><br>3. Kanis, J.A. Assessment of Fracture Risk and Its Application to Screening for Postmenopausal Osteoporosis: Synopsis of a WHO Report. Osteoporos. Int. 1994, 4, 368–381. https://doi.org/10.1007/bf01622200.<br><br>4. Cruz-Jentoft AJ, Bahat G, Bauer J, Boirie Y, Bruyère O, Cederholm T, et al. Sarcopenia: revised European consensus on definition and diagnosis. Age and Ageing. 2018 Sep 24;48(1):16–31 (doi: 10.1093/ageing/afy169, PMID: 30312372) |

| | |
|---|---|
| **Gait speed (GS)** was performed once and assessed over ground at a fast pace using 4-meter straight walking trajectory with a standing start protocol. Timing[a] was started when the first foot movement was observed and was ceased once the subject had completely crossed the end line. Gait speed was expressed in m/s. Normal values are > 0.8 m/sec [4].<br><br>**Timed Up and Go test (TUG)** recorded how long it took for a subject to rise from a standard height chair, walk 3 m at a comfortable pace to a mark placed on the floor, turn around, walk back to the starting point, and sit back down on the chair. TUG was expressed in seconds. Normal values < 20 sec [4].<br><br>**Tinetti Assessment Tool/Test (TT)** is a physical performance examination that scores specific gait and balance-related tasks on a three-point ordinal scale with a range of 0 to 2, where 0 represents the most impairment and 2 represents independence. Maximum score is 28 points (12 for gait and 16 for balance). Subjects below 19 and in the range of 19-24 or 25-28 points are at high and medium risk or low fall risk, respectively [4]. | |

**Table S3. Genotype phenotype correlation of SNVs rs8057044 (G/A) in OP.** Numerical variable were compared using and Mann-Whitney U test (GA/(AA-GG) for over-dominant model and Kruskal-Wallis test (AA/GA/GG) test for additive model. Nominal variables were tested by χ2. Trend values (0.05 < P-value ⩾ 0.10) are indicated italics.

| SNV rs8057044 | | | | | | | |
|---|---|---|---|---|---|---|---|
| **Parameter** | **GA** n = 60 | **AA-GG** n = 60 | **P**$^a$ Mann-Whitney | **AA** n = 27 | **GA** n = 60 | **GG** n = 33 | **P** Kruskal-Wallis |
| Age (years) | 66.6±0.8 | 66.1±0.7 | NS | 65.5±0.8 | 66.2±0.8 | 67.9±1.1 | NS |
| BMI (kg/m2) | 25.2±0.4 | 27.2±0.4 | **0.0014** | 26.6±0.5 | 25.1±0.5 | 28.1±0.6 | **0.0004*** |
| Waist (cm) | 90.7±1.0 | 94.1±1.0 | **0.0196** | 91.9±1.2 | 90.5±1.1 | 97.2±1.4 | **0.0007*** |
| Obesity-BMI$^{30}$ (%) | 16.6 | 25.0 | NS | 29.6 | 16.6 | 21.2 | NS |
| Fasting Glucose (mmol/L) | 5.3±0.1 | 5.4±0.1 | NS | 5.3±0.1 | 5.35±0.8 | 5.4±0.1 | NS |
| Fasting insulin (µU/mL) | 8.0±0.5 | 8.6±0.6 | NS | 8.1±0.5 | 8.3±0.6 | 8.4±1.0 | NS |
| HOMA-IR | 1.97±0.1 | 2.2±0.2 | NS | 2.0±0.1 | 2.1±0.1 | 2.1±0.2 | NS |
| Insulin resistance (%) | 31.6 | 33.3 | NS | 29.6 | 31.6 | 36.4 | NS |
| SBP (mmHg) | 120.8±1.3 | 123.3±1.9 | NS | 127.6±2.8 | 120.8±1.2 | 119.7±2.5 | **0.0136**** |
| DBP (mmHg) | 74.1±0.8 | 77.3±1.1 | **0.018** | 79.3±1.7 | 74.2±0.8 | 75.7±1.3 | **0.0022**** |
| HbA1c (%) | 5.46±0.0 | 5.56±0.0 | NS | 5.4±0.0 | 5.5±0.0 | 5.5±0.1 | NS |
| TG (mmol/L) | 1.15±0.0 | 1.12±0.0 | NS | 1.1±0.0 | 1.1±0.05 | 1.2±0.1 | NS |
| HDL-C (mmol/L) | 1.58±0.0 | 1.48±0.0 | **0.0197** | 1.4±0.0 | 1.57±0.0 | 1.5±0.0 | **0.0227**** |
| Central obesityWaist (%) | 58.3 | 76.7 | **0.0024** | 85.1 | 58.3 | 69.7 | **0.0022**** |
| Hyperglycemia (%) | 35.0 | 35.0 | NS | 37.0 | 35.0 | 33.3 | NS |
| Hypertension (%)c | 40.0 | 40.0 | NS | 40.7 | 40.0 | 39.4 | NS |
| High TG (%) | 20.0 | 16.6 | NS | 18.5 | 20.0 | 15.1 | NS |
| Low HDL-C (%) | 28.3 | 31.6 | NS | 14.8 | 28.3 | 45.4 | 0.001 |
| MetS$^{ATPIII}$ (%) | 30.0 | 31.6 | NS | 22.2 | 30.0 | 39.4 | NS |
| CRP (mmol/L) | 28.6±0.0 | 38.1±0.0 | **0.047** | 0.4±0.1 | 0.28±0.0 | 0.38±0.0 | **0.0209**** |
| Severe-OP (%) | 53.3 | 36.7 | **0.0095** | 37.0 | 53.3 | 36.4 | **0.0344**** |
| BMD LS (g/cm$^2$)$^b$ | 0.90±0.1 | 0.93±0.01 | **0.0017** | 0.949±0.0 | 0.901±0.0 | 0.92±0.0 | **0.0004****<br>*0.0539**** |
| BMD HIP (g/cm$^2$) | 0.95±0.01 | 0.98±0.01 | **0.0014** | 0.989±0.0 | 0.952±0.0 | 0.967±0.0 | **0.0003****<br>*0.0539* *** |
| BMD FN (g/cm$^2$) | 0.79±0.01 | 0.81±0.01 | **0.0014** | 0.826±0.0 | 0.787±0.0 | 0.804±0.0 | **0.0003****<br>*0.0539**** |
| BMD RA (g/cm$^2$) | 0.64±0.0 | 0.65±0.0 | **0.0022** | 0.652±0.0 | 0.638±0.0 | 0.642±0.0 | **0.0001****<br>0.159*** |
| TBS L1-L4 | 1.21±0.0 | 1.24±0.0 | **0.0192** | 1.23±0.0 | 1.22±0.0 | 1.25±0.0 | NS |
| Osteocalcin (ng/ml) | 19.3±1.1 | 18.3±0.8 | NS | 19.7±1.0 | 19.24±1.2 | 16.5±1.1 | **0.0393***** |
| P1NP (ng/ml) | 44.0±2.7 | 42.3±2.5 | NS | 46.2±1.0 | 43.9±2.8 | 37.2±4.0 | **0.0627***** |
| Beta-crosslaps (ng/ml) | 0.37±0.0 | 0.36±0.0 | NS | 0.4±0.0 | 0.39±0.0 | 0.27±0.0 | **0.0015*****<br>**0.0059*** |
| SUM$^{stat}$ (%) | 53.3 | 36.6 | **0.0095** | 40.7 | 53.3 | 33.3 | **0.0248**** |
| Grip strength right (kg) | 20.29±0.4 | 21.63±0.4 | **0.0208** | 20.7±0.4 | 20.6±0.4 | 22.1±0.7 | *0.0682** |
| Grip strength left (kg) | 18.37±0.3 | 19.19±0.4 | NS | 18.4±0.5 | 18.9±0.4 | 19.04±0.7 | NS |
| CST (sec) | 16.5±0.6 | 14.5±0.5 | **0.0153** | 14.6±0.7 | 16.5±0.7 | 14.4±0.7 | **0.0390*** |
| Gait speed (m/sec) | 1.1±0.0 | 1.1±0.0 | NS | 1.1±0.0 | 1.1±0.0 | 1.1±0.0 | NS |
| TUG (sec) | 12.6±0.6 | 12.02±0.4 | NS | 12.0±0.5 | 12.2±0.6 | 12.9±0.6 | NS |
| TINETTI score (points) | 23.0±0.2 | 23.4±0.2 | NS | 23.3±0.4 | 23.0±0.2 | 23.40.3 | NS |
| FRAX PLUS major (%) | 10.2±0.5 | 9.32±0.5 | NS | 9.6±0.6 | 10.0±0.4 | 9.3±0.8 | NS |
| FRAX PLUS hip (%) | 3.3±0.3 | 2.9±0.3 | NS | 2.8±0.3 | 3.2±0.3 | 3.0±0.6 | NS |

a, NS stands for non-significant; b, BMD for LS (lumbar spine), FN (femoral neck), RA (33% radius); All BMD values were adjusted for BMI. MetS, Metabolic syndrome; P1NP, Procollagen type I N-terminal propeptide; TBS, trabecular bone score; SUMstat muscle, summary statistics of muscular tests. *, stands for significance between GA and GG; ** stands for significance between AA and GA; ***, stands for significance between AA and GG;

**Table S4. Genotype phenotype correlation of SNV rs9939609 (T/A) in osteoporotic patients.** Numerical variable were compared using Kruskal-Wallis test (AA/TA/TT) and Mann-Whitney U test (TA/(AA-TT) while nominal variables were tested by $\chi^2$. Trend values (0.05 < P-value ⩾ 0.10) are indicated in italics.

| SNV rs9939609 | | | | | | | |
|---|---|---|---|---|---|---|---|
| **Parameter** | **TA** n = 56 | **AA-TT** n = 64 | **P** Mann-Whitney | **AA** n = 24 | **TA** n = 56 | **TT** n = 40 | **P** Kruskal-Wallis |
| Age (years) | 66.3±0.8 | 66.4±0.7 | NS | 67.1±1.2 | 66.3±0.8 | 66.0±0.8 | NS |
| BMI (kg/m$^2$) | 25.3±0.5 | 26.9±0.4 | **0.0108** | 27.8±0.7 | 25.3±0.5 | 26.5±0.5 | **0.0043*** |
| Waist (cm) | 91.0±1.1 | 93.6±0.9 | *0.084* | 96.8±1.5 | 91.0±1.15 | 91.7±1.2 | **0.0050*** **0.0098**** |
| Obesity-BMI$^{30}$ (%)[b] | 16.1 | 25.0 | *0.0893* | 29.2 | 16.1 | 22.5 | NS |
| Fasting Glucose (mmol/L) | 5.32±0.1 | 5.42±0.07 | NS | 5.4±0.1 | 5.3±0.1 | 5.4±0.1 | NS |
| Fasting insulin (µU/mL) | 8.5±0.6 | 8.04±0.5 | NS | 7.4±0.8 | 8.5±0.6 | 8.4±0.6 | NS |
| HOMA-IR[c] | 2.13±0.2 | 2.015±0.1 | NS | 1.85±0.2 | 2.1±0.2 | 2.1±0.16 | NS |
| Insulin resistance (%)[d] | 32.1 | 32.8 | NS | 20.8 | 32.1 | 40.0 | *0.0806* |
| SBP (mmHg)[e] | 121.4±1.4 | 122.69±0.8 | NS | 128.3±2.8 | 121.4±1.4 | 119.2±2.1 | **0.0181*** **0.0126**** |
| DBP (mmHg) | 75.0±0.9 | 76.4±0.9 | NS | 78.5±1.7 | 75.0±0.9 | 75.1±1.2 | *0.0505** |
| HbA1c (%) | 5.5±0.0 | 5.52±0.0 | NS | 5.6±0.1 | 5.5±0.04 | 5.5±0.0 | NS |
| TG (mmol/L) | 1.16±0.05 | 1.11±0.0 | NS | 1.12±0.1 | 1.16±0.0 | 1.0±0.0 | NS |
| HDL-C (mmol/L) | 1.56±0.03 | 1.50±0.0 | NS | 1.56±0.0 | 1.56±0.0 | 1.46±0.0 | **0.0402**** |
| Central obesityWaist (%)[b] | 58.9 | 75.0 | **0.0080** | 83.3 | 58.9 | 70.0 | **0.0088*** |
| Hyperglycemia (%)c | 35.7 | 34.4 | NS | 37.5 | 35.7 | 32.5 | NS |
| Hypertension (%)c | 37.5 | 42.2 | NS | 37.5 | 37.5 | 45.0 | NS |
| High TG (%)c | 23.2 | 14.0 | *0.0676* | 16.6 | 23.2 | 12.5 | NS |
| Low HDL-C (%)c | 26.7 | 32.8 | NS | 16.6 | 26,7 | 42.5 | **0.0051***** |
| MetS$^{ATPIII}$ (%) | 30.35 | 31.2 | NS | 20.8 | 30.35 | 37.5 | NS |
| CRP mmol/L | 0.29±0.03 | 0.406±0.04 | **0.0316** | 0.37±0.0 | 0.29±0.0 | 0.42±0.1 | **0.0393***** |
| Severe-OP with fractures (%) | 55.35 | 35.9 | **0.0026** | 33.3 | 55.35 | 37.5 | **0.0092***** |
| BMD LS (g/cm$^2$) | 0.90±0.01 | 0.93±0.01 | **0.0127** | 0.94±0.01 | 0.907±0.0 | 0.921±0.01 | **0.0050** * |
| BMD HIP (g/cm$^2$) | 0.95±0.01 | 0.97±0.0 | **0.0108** | 0.985±0.01 | 0.954±0.0 | 0.968±0.01 | **0.0043*** |
| BMD FN (g/cm$^2$) | 0.79±0.01 | 0.81±0.01 | **0.0108** | 0.822±0.01 | 0.789±0.0 | 0.804±0.01 | **0.0043*** |
| BMD RA (g/cm$^2$) | 0.64±0.0 | 0.64±0.0 | **0.0179** | 0.65±0.0 | 0.63±0.0 | 0.64±0.0 | **0.0026*** **0.0392**** |
| TBS L1-L4 | 1.21±0.0 | 1.24±0.0 | *0.0883* | 1.26±0.02 | 1.21±0.01 | 1.23±0.01 | **0.0135*** |
| Osteocalcin (ng/ml) | 19.26±1.2 | 18.4±0.7 | NS | 16.6±1.2 | 19.26±1.2 | 19.4±0.95 | *0.0629*** |
| P1NP (ng/ml) | 43.6±2.9 | 42.7±2.3 | NS | 37.4±4.3 | 43.6±2.9 | 45.9±2.6 | *0.0802*** |
| Beta-crosslaps (ng/ml) | 0.39±0.03 | 0.35±0.02 | NS | 0.26±0.02 | 0.39±0.0 | 0.39±0.0 | **0.0063*** **0.0014**** |
| SUM$^{stat}$ muscular alterations (% | 51.7 | 39.1 | **0.0481** | 45.8 | 51.7 | 35.0 | *0.0696* |
| Grip strength right (kg) | 20.54±0.4 | 21.33±0.4 | NS | 22.17±0.8 | 20.54±0.4 | 20.84±0.4 | *0.0519* * |
| Grip strength left (kg) | 18.9±0.3 | 18.7±0.0 | NS | 19.1±0.7 | 18.8±0.3 | 18.49±0.5 | NS |
| CST (sec) | 16.2±0.7 | 14.9±0.5 | NS | 15.1±0.7 | 16.2±0.7 | 14.8±0.6 | NS |
| Gait speed (m/sec) | 1.1±0.0 | 1.1±0.0 | NS | 1.0±0.0 | 1.1±0.0 | 1.1±0.0 | NS |
| TUG (sec) | 12.48±0.6 | 12.19±0.4 | NS | 12.6±0.6 | 12.48±0.6 | 11.9±0.5 | NS |
| TINETTI score (points) | 23.0±0.3 | 23.4±0.2 | NS | 23.3±0.5 | 23.0±0.3 | 23.4±0.2 | NS |
| FRAX PLUS major (%) | 10.03±0.5 | 9.5±0.5 | NS | 9.32±0.9 | 10.03±0.5 | 9.69±0.6 | NS |
| FRAX PLUS hip (%) | 3.2±0.3 | 3.03±0.3 | NS | 3.1±0.6 | 3.2±0.3 | 2.98±0.3 | NS |

a, NS stands for non-significant; b, BMD for LS (lumbar spine), FN (femoral neck), RA (33% radius); All BMD values were adjusted for BMI. MetS, Metabolic syndrome; P1NP, Procollagen type I N-terminal propeptide; TBS, trabecular bone score; SUM$^{stat}$ muscle, summary statistics for muscular tests. *, stands for significance between AA and TA; **, stands for significance between AA and TT; ***, stands for significance between TA and TT

**Table S5. Genotype phenotype correlation of SNVs rs9930506 (G/A) in OP.** Numerical variable were compared using Kruskal-Wallis test (AA/GA/GG) and Mann-Whitney U test (GA/(AA-GG) while nominal variables were tested by $\chi^2$. Trend values (0.05 < P-value ⩾ 0.10) are indicated in italics.

## SNV rs9930506

| Parameter | GA<br>n = 56 | AA-GG<br>n = 59 | P<br>Mann-Whitney | AA<br>n = 34 | GA<br>n = 56 | GG<br>n = 25 | P<br>Kruskal-Wallis |
|---|---|---|---|---|---|---|---|
| Age (years) | 66.0±0.8 | 66.7±0.7 | NS | 66.35±0.92 | 66.0±0.8 | 67.1±1.1 | NS |
| BMI (kg/m$^2$) | 25.4±0.4 | 27.1±0.4 | **0.0056** | 26.4±0.6 | 25.4±0.4 | 28.1±0.7 | **0.0011***<br>_0.07**_ |
| Waist (cm) | 90.7±1.0 | 94.2±1.1 | **0.0197** | 91.9±1.3 | 90.7±1.0 | 97.2±1.7 | **0.0010***<br>**0.0172**** |
| Obesity-BMI$^{30}$ (%)$^b$ | 14.7 | 27.1 | **0.0184** | 23.5 | 14.7 | 32.0 | **0.0331** |
| Fasting Glucose (mmol/L) | 5.4±0.1 | 5.3±0.1 | NS | 5.3±0.1 | 5.4±0.1 | 5.4±0.1 | NS |
| Fasting insulin (µU/mL) | 8.7±0.6 | 7.8±0.4 | NS | 7.9±0.6 | 8.7±0.6 | 7.6±0.8 | NS |
| HOMA-IR$^c$ | 2.2±0.2 | 1.9±0.0 | NS | 1.95±0.2 | 2.2±0.2 | 1.89±0.2 | NS |
| Insulin resistance (%)$^d$ | 37.3 | 27.1 | _0.08_ | 32.3 | 37.7 | 20.0 | _0.079_ |
| SBP (mmHg)$^e$ | 119.8±1.4 | 124.3±1.8 | _0.051_ | 119.8±2.3 | 119.8±1.4 | 130.3±2.6 | **0.0003***<br>**0.0038**** |
| DBP (mmHg) | 74.1±0.9 | 77.4±1.0 | **0.0138** | 76.1±1.3 | 74.1±0.9 | 79.2±1.6 | **0.0034*** |
| HbA1c (%) | 5.49±0.0 | 5.5±0.0 | NS | 5.5±0.0 | 5.4±0.0 | 5.5±0.1 | NS |
| TG (mmol/L) | 1.5±0.0 | 1.12±0.0 | NS | 1.1±0.0 | 1.15±0.0 | 1.16±0.1 | NS |
| HDL-C (mmol/L) | 1.6±0.0 | 1.5±0.0 | NS | 1.47±0.0 | 1.54±0. | 1.57±0.0 | _0.07**_ |
| Central obesityWaist (%)$^b$ | 60.7 | 74.6 | **0.021** | 70.6 | 60.6 | 80.0 | **0.039** |
| Hyperglycemia (%)c | 37.7 | 32.2 | NS | 35.3 | 37.7 | 32.0 | NS |
| Hypertension (%)c | 34.4 | 45.8 | _0.07_ | 44.1 | 34.4 | 48.0 | NS |
| High TG (%)c | 19.7 | 16.9 | NS | 14.7 | 19.6 | 20.0 | NS |
| Low HDL-C (%)c | 31.14 | 28.8 | NS | 41.1 | 31.1 | 12.0 | **0.0027** |
| MetS$^{ATPIII}$ (%) | 31.14 | 30.5 | NS | 38.2 | 31.14 | 20.0 | NS |
| CRP (mmol/L) | 26.7±0.0 | 40±0.0 | **0.0074** | 0.43±0.1 | 0.28±0.0 | 0.40±0.0 | **0.0120***<br>**0.0190***** |
| Severe-OP with fractures (%) | 52.5 | 37.3 | **0.018** | 38.2 | 52.4 | 36.0 | _0.0597_ |
| BMD LS (g/cm$^2$) | 0.90±0.01 | 0.93±0.01 | **0.0046** | 0.92±0.01 | 0.903±0.0 | 0.947± 0.0 | **0.0013*** |
| BMD HIP (g/cm$^2$) | 0.95±0.0 | 0.98±0.01 | **0.0056** | 0.967±0.0 | 0.954±0.0 | 0.98±0.0 | **0.0011*** |
| BMD FN (g/cm$^2$) | 0.79±0.01 | 0.81±0.01 | **0.0056** | 0.803±0.0 | 0.799±0.0 | 0.825±0.0 | **0.0011*** |
| BMD RA (g/cm$^2$) | 0.64±0.0 | 0.65±0.01 | **0.0085** | 0.642±0.0 | 0.639±0.0 | 0.652±0.0 | **0.0006***<br>**0.0231**** |
| TBS L1-L4 | 1.23±0.0 | 1.23±0.01 | NS | 1.22±0.0 | 1.22±0.0 | 1.26±0.0 | **0.0397***<br>0.0698** |
| Osteocalcin (ng/ml) | 19.07±1.1 | 18.5±0.9 | NS | 18.5±1.0 | 19.1±1.1 | 18.54±1.6 | NS |
| P1NP (ng/ml) | 42.8±2.6 | 43.6±2.6 | NS | 43.2±2.9 | 42.8±2.5 | 44.04±4.8 | NS |
| Beta-crosslaps (ng/ml) | 0.38±0.02 | 0.35±0.02 | NS | 0.37±0.3 | 0.38±0.0 | 0.33±0.0 | NS |
| SUM$^{stat}$ (%) | 49.2 | 40.7 | NS | 32.3 | 49.2 | 48.0 | NS |
| Grip strength right (kg) | 20.6±0.4 | 21.3±0.4 | NS | 20.7±0.4 | 20.6±0.3 | 22.15±0.8 | _0.0525*_ |
| Grip strength left (kg) | 18.2±0.3 | 18.75±0.4 | NS | 18.36±0.4 | 18.8±0.3 | 19.3±0.7 | NS |
| CST (sec) | 15.9±0.4 | 15.9±0.6 | NS | 14.8±0.6 | 15.9±0.6 | 15.4±0.7 | NS |
| Gait Speed (m/sec) | 1.1±0.0 | 1.00.0 | NS | 1.1±0.0 | 1.10.0 | 1.0±0.0 | NS |
| TUG (sec) | 11.9±0.6 | 12.7±0.4 | NS | 12.3±0.6 | 11.9±0.6 | 13.3±0.7 | NS |
| TINETTI score | 23.2±0.3 | 23.2±0.2 | NS | 23.3±0.3 | 23.2±0.3 | 23.1±0.4 | NS |
| FRAX PLUS major (%) | 10.4±0.5 | 9.1±0.4 | _0.075_ | 9.7±0.6 | 10.38±0.5 | 8.26±0.4 | **0.0177*** |
| FRAX PLUS hip (%) | 3.56±0.4 | 5.28±0.2 | **0.034** | 7.69±0.3 | 3.56±0.36 | 2.1±0.2 | **0.0161***<br>**0.0457**** |

a, NS stands for non-significant; b, BMD for LS (lumbar spine), FN (femoral neck), RA (33% radius); All BMD values were adjusted for BMI. MetS, Metabolic syndrome; CTS, chair stand test; P1NP, Procollagen type I N-terminal propeptide; TBS, trabecular bone score; SUM$^{stat}$ muscle, summary statistics for muscular tests. *, stands for significance between GA and GG; **, stands for significance between AA and GG; ***, stands for significance between AA and GA;

**Table S6. Genotype phenotype correlation of haplotype pairs of the FTO gene in OP.** Data are presented as mean ± SEM. Numerical variable were compared using Kruskal-Wallis and Mann-Whitney U tests while nominal variables were tested by $\chi^2$. Both significant and non-significant values were included. Trend values (0.05 < P-value ≥ 0.10) are indicated in italics

| **Parameter** | **Homozygous H1/H1** | **Homozygous H9/H9** | **Heterozygous H1/H9** | **P** Kruskal-Wallis | **P** Mann-Whitney U | **P ANOVA (α)** |
|---|---|---|---|---|---|---|
| Age (years) | 65.5±1.0 | 67.0±1.2 | 65.9±0.9 | NS | NS | NS |
| BMI (kg/$m^2$) | 26.5±0.6 | 28.1±0.7 | 24.7±0.5 | **0.0002** | **0.0188*** <br> **0.0001**** | **0.0002** <br> (0.98) |
| Waist (cm) | 91.8±1.5 | 97.2±1.6 | 89.6±1.1 | **0.0026** | **0.0002**** <br> **0.0145*** | **0.0009** <br> (0.94) |
| Obesity-BMI (%)[b] | 21.42 | 30.23 | 12.24 | **0.0351** | NA | NA |
| Fasting Glucose (mmol/L) | 5.2±0.1 | 5.5±0.1 | 5.3±0.1 | NS | *0.0788** | NS |
| Fasting insulin (μU/mL) | 7.3±0.6 | 7.9±0.9 | 7.4±0.5 | NS | NS | NS |
| HOMA-IR[c] | 1.8±0.2 | 1.9±0.2 | 1.8±0.1 | NS | NS | NS |
| Insulin resistance (%) | 28.57 | 23.25 | 28.57 | NS | NS | NS |
| SBP (mmHg)[e] | 119.8±2.8 | 130.2±3.0 | 120.7±1.4 | **0.0412** | **0.0017**** | **0.0057** <br> (0.84) |
| DBP (mmHg) | 76.4±1.5 | 79.2±1.9 | 74.1±0.9 | NS | **0.0069**** | **0.0277** <br> (0.66) |
| HbA1c (%) | 5.5±0.0 | 5.6±0.1 | 5.5±0.0 | NS | NS | NS |
| TG (mmol/L) | 1.1±0.1 | 1.2±0.1 | 1.1±0. | NS | NS | NS |
| HDL-C (mmol/L) | 1.4±0.0 | 1.5±0.0 | 1.5±0.0 | *0.0622* | **0.0184*** <br> **0.0172***** | **0.0313** <br> (0.64) |
| Central obesityWaist (%) | 71.42 | 86.04 | 55.10 | **0.0011** | NA | NA |
| Hyperglycemia (%)c | 32.14 | 37.21 | 34.69 | NS | NA | NA |
| Hypertension (%)c | 39.28 | 41.86 | 34.69 | NS | NA | NA |
| High TG (%)c | 14.28 | 18.60 | 20.40 | NS | NA | NA |
| Low HDL -C(%)c | 46.42 | 9.30 | 28.57 | **0.0003** | NA | NA |
| MetS$^{ATPIII}$ | 39.3 | 18.60 | 28.56 | *0.0789* | NA | NA |
| CRP (mg/dl) | 38.1±9.52 | 38.1±0.0 | 28.6±0.0 | **0.0123** | **0.0298***** <br> *0.0747** | *0.0586* <br> (0.54) |
| Severe OP with fractures (%) | 35.71 | 32.55 | 53.06 | **0.0285** | NA | NA |
| BMD LS (g/$cm^2$) | 0.92±0.01 | 0.94±0.01 | 0.89±0.01 | **0.0003** | **0.0001**** <br> **0.0208***** | **0.0002** |
| BMD HIP (g/$cm^2$) | 0.97±0.01 | 0.99±0.01 | 0.94±0.01 | **0.0002** | **0.0001**** <br> **0.0188***** | **0.0002** <br> (0.98) |
| BMD FN (g/$cm^2$) | 0.80±0.01 | 0.82±0.01 | 0.79±0.01 | **0.0002** | **0.0001**** <br> **0.0188***** | **0.0002** <br> (0.98) |
| BMD RA (g/$cm^2$) | 0.64±0.00 | 0.65±0.00 | 0.63±0.00 | **< 0.0001** | **0.0001**** <br> **0.0206*** <br> **0.0423***** | **< 0.0001** <br> (0.99) |
| TBS L1 – L4 | 1.23±0.02 | 1.26±0.02 | 1.22±0.01 | NS | *0.0501*** | NS |
| Osteocalcin (ng/ml) | 17.82±1.11 | 16.82±1.25 | 18.09±1.19 | NS | NS | NS |
| P1NP (ng/ml) | 42.19±3.39 | 39.32±4.72 | 40.90±2.89 | NS | NS | NS |
| Beta-crosslaps (ng/ml) | 0.37±0.03 | 0.70±0.03 | 0.35±0.02 | NS | **0.0338**** <br> **0.0306*** | *0.0739* <br> (0.51) |
| SUM$^{stat}$ (%) | 32.14 | 46.31 | 51.02 | *0.0738* | NA | NA |
| Grip strength right (kg) | 20.96±0.56 | 22.57±0.83 | 20.56±0.45 | NS | **0.0223**** | *0.0584* |

| | | | | | | |
|---|---|---|---|---|---|---|
| Grip strength left (kg) | 18.94±0.64 | 19.37±0.77 | 18.71±0.37 | NS | NS | NS |
| Chair Stand Test (sec) | 14.64±0.73 | 15.35±0.77 | 16.08±0.74 | NS | NS | NS |
| Gait speed (m/sec) | 1.13±0.04 | 1.303±0.04 | 1.14±0.03 | NS | NS | NS |
| TUG (sec) | 12.09±0.66 | 13.13±0.68 | 12.20±0.71 | *0.0627* | NS | NS |
| TINETTI test (points) | 23.4±0.3 | 23.2±0.5 | 23.0±0.3 | NS | NS | NS |
| FRAX PLUS major (%) | 8.82±0.63 | 8.42±0.49 | 9.84±0.49 | NS | NS | NS |
| FRAX PLUS hip (%) | 2.69±0.36 | 2.23±0.19 | 3.28±0.31 | NS | **0.0326**** | NS |

a, NS stands for non-significant and NA for non applicable; b, Obesity was considered based on BMI > 30 kg/m2; c, SBP and DBP, stand for systolic and diastolic blood pressure; * indicates significance between H1/H1 and H9/H9; **, indicates significance between H1/9 and H9/H9; MetS, Metabolic syndrome; P1NP, Procollagen type I N-terminal propeptide; TBS, trabecular bone score; $SUM^{stat}$, summary statistics for muscular tests.

**Table S7. Features of non-osteoporotic (non-OP) and osteoporotic (OP) patients.** Subjects were classified according to AACE and severe OP by WHO criteria. Data are presented as mean ± SD. Groups were compared using Mann-Whitney U test (numerical variables) and $\chi^2$ (nominal variables).

| Parameter | Non-OP | OP | $P^a$ value |
|---|---|---|---|
| *n* | 68 | 120 | NA |
| Age (years) | 60.7 ± 8.5 | 66.4 ± 8.2 | **0.0001** |
| BMI (kg/m$^2$) | 30.6 ± 5.3 | 26.2 ± 5.0 | **0.0001** |
| Waist (cm) | 99.5 ± 11.2 | 92.4 ± 11.6 | **0.0001** |
| Obesity$^{BMI}$ (%)$^b$ | 48.5 | 20.8 | **0.0001** |
| Fasting glucose (mmol/L) | 5.6 ± 1.0 | 5.4 ± 0.8 | NS |
| Fasting insulin (μU/mL) | 9.8 ± 5.9 | 8.3 ± 5.9 | NS |
| HOMA$_{IR}$$^c$ | 2.6 ± 3.0 | 2.1 ± 1.7 | NS |
| Insulin resistance (%)$^d$ | 35.3 | 32.5 | NS |
| SBP (mm Hg)$^e$ | 125.8 ± 14.6 | 122.1 ± 17.8 | NS |
| DBP (mm Hg)$^e$ | 77.7 ± 9.9 | 75.8 ± 10.4 | NS |
| HbA1c (%) | 5.7 ± 0,7 | 5.5 ± 0,4 | **0.0057** |
| TG (mmol/L) | 1.2 ± 0.6 | 1.13 ± 0.5 | NS |
| HDL-C (mmol/L) | 1.54 ± 0.4 | 1.53 ± 0.3 | NS |
| Central obesity (%) | 86.8 | 67.5 | **0.020** |
| Hyperglycemia (%) | 19.1 | 18.3 | **0.032** |
| Hypertension (%) | 57.3 | 40.0 | **0.027** |
| High TG (%) | 19.1 | 18.3 | *0.056* |
| Low HDL-C (%) | 44.1 | 30 | NS |
| MetS$^{ATPIII}$ (%) | 51.5 | 30.8 | **0.031** |
| CRP (mmol/L) | 50.5 ± 66.6 | 33.3 ± 38.1 | **0.039** |
| Osteopenia (%) | 51.5 | 2.5 | **0.0001** |
| Severe-OP with fractures (%) | 0.0 | 48.3 | NA |
| BMD lumbar spine (g/cm$^2$) | 1.1 ± 0.1 | 0.9 ± 0.1 | NA |
| BMD hip (g/cm$^2$) | 1.0 ± 0.1 | 0.8 ± 0.1 | NA |
| BMD femoral neck (g/cm$^2$) | 0.9 ± 0.1 | 0.76 ± 0.1 | NA |
| BMD radius 33% (g/cm$^2$) | 0.7 ± 0.1 | 0.55 ± 0.1 | NA |
| TBS L1 – L4 | 1.3 ± 0.1 | 1.2 ± 0.1 | **0.0001** |
| Osteocalcin (ng/ml) | 18.8 ± 6.9 | 19.0 ± 10.8 | NS |
| P1NP (ng/ml) | 47.2 ± 19.3 | 43.2 ± 28.6 | **0.02** |
| Beta-crosslaps (ng/ml) | 0.4 ± 0.1 | 0.4± 0.2 | NS |
| Fibrinogen (g/L) | 3.9 ± 0.7 | 3.7 ± 0.6 | **0.0221** |
| Uric acid (mmol/L) | 0.29 ± 0.07 | 0.26 ± 0.06 | **0.0197** |
| Magnesium (mmol/L) | 0.089 ± 0.0 | 0.093 ± 0.0 | NS |
| Calcium (mmol/L) | 1.0 ± 0.0 | 1.0 ± 0.0 | NS |
| Vitamin D (ng/ml) | 28.2 ± 10.5 | 29.8 ± 10.2 | NS |
| PTH (pg/ml) | 40.0 ± 15.1 | 43.4 ± 18.0 | NS |
| ALP (U/L) | 66.3 ± 20.2 | 65.1 ± 20.3 | NS |
| SUM$^{stat}$ muscle (%) | 22.7 | 45.0 | **0.0083** |
| Grip strength right (kg) | 24.2 ± 5.3 | 20.9 ± 4.5 | **0.0001** |
| Grip strength left (kg) | 21.8 ± 4.8 | 18.8 ± 4.2 | **0.0001** |
| CST (sec) | 12.7 ± 4.2 | 15.5 ± 6.3 | **0.0015** |
| Gait speed (m/sec) | 1.3 ± 0.3 | 1.1 ± 0.3 | **0.0001** |
| TUG (sec) | 10.1 ± 3.4 | 12.3 ± 5.7 | **0.0046** |
| TINETTI test (points) | 24.2 ± 2.1 | 23.2 ± 2.8 | **0.0163** |

a, NS stands for non-significant and NA for non-applicable; b, Obesity was considered based on BMI ≥ 30 kg/m$^2$; c, HOMA-IR was calculated as glucose (mmol/L) x Insulin (μU/ml)/22.5; d, insulin resistance was considered as function of HOMA$_{IR}$ values with cut-off 1.92 as mean + 2 SEM of lean non-OP subjects; e, SBP and DBP, stand for systolic and diastolic blood pressure, respectively; MetS, Metabolic syndrome; P1NP, Procollagen type I N-terminal propeptide; TBS, trabecular bone score; SUM$^{stat}$, summary statistics of muscular tests.
